# Nanopore Sequencing Indicates That Tandem Amplification of Chromosome 20q11.21 in Human Pluripotent Stem Cells Is Driven by Break-Induced Replication

**DOI:** 10.1089/scd.2021.0013

**Published:** 2021-05-25

**Authors:** Jason A. Halliwell, Duncan Baker, Kim Judge, Michael A. Quail, Karen Oliver, Emma Betteridge, Jason Skelton, Peter W. Andrews, Ivana Barbaric

**Affiliations:** ^1^ Department of Biomedical Science, University of Sheffield, Sheffield, United Kingdom.; ^2^ Sheffield Diagnostic Genetic Services, Sheffield Children's Hospital, Sheffield, United Kingdom.; ^3^ Department of Sequencing R & D, Wellcome Sanger Institute, Hinxton, United Kingdom.

**Keywords:** microhomology-mediated break-induced replication, genetic instability, embryonic stem cells, induced pluripotent stem cells, Oxford Nanopore, Chromosome 20

## Abstract

Copy number variants (CNVs) are genomic rearrangements implicated in numerous congenital and acquired diseases, including cancer. The appearance of culture-acquired CNVs in human pluripotent stem cells (PSCs) has prompted concerns for their use in regenerative medicine. A particular problem in PSC is the frequent occurrence of CNVs in the q11.21 region of chromosome 20. However, the exact mechanism of origin of this amplicon remains elusive due to the difficulty in delineating its sequence and breakpoints. Here, we have addressed this problem using long-read Nanopore sequencing of two examples of this CNV, present as duplication and as triplication. In both cases, the CNVs were arranged in a head-to-tail orientation, with microhomology sequences flanking or overlapping the proximal and distal breakpoints. These breakpoint signatures point to a mechanism of microhomology-mediated break-induced replication in CNV formation, with surrounding *Alu* sequences likely contributing to the instability of this genomic region.

## Introduction

Copy number variants (CNVs) are gains or losses of DNA segments ranging in size from ∼50 bp to several megabases [1]. By affecting the dosage of genes and regulatory regions within the amplified or deleted sequence, CNVs underpin the etiology of many diseases from developmental disorders to cancer [1]. The profound effect of CNV acquisition on cellular phenotype has also been described in human pluripotent stem cells (PSCs), which frequently gain a CNV located on chromosome 20 in the region q11.21 upon prolonged culture [2–5]. Once gained, the chromosome 20q11.21 CNV bestows on the variant PSC a growth advantage due to resistance to apoptosis [5,6]. Since the same CNV is a genomic hallmark of some cancers [7], it represents a potential impediment to the use of PSC in regenerative medicine.

The chromosome 20q11.21 CNV is typically gained as a tandem duplication, although PSC lines with four or five copies of this CNV have been reported [2,8]. The length of the duplicated region is also variable between different lines and ranges from 0.6 to 4 Mb [2,8]. Nonetheless, the shared region common to all of the reported variants contains a dosage-sensitive antiapoptotic gene, *BCL2L1*, which has been identified as the driver gene, overexpression of which is responsible for the selective growth advantage of variant PSC carrying this CNV [5,6,8]. However, the nature of the mutational events that generate these chromosome 20q11.21 CNVs has not been elucidated in PSCs.

CNVs can be generated by a number of different aberrations that may occur during DNA synthesis or repair [7], and may be distinguished by the characteristics of the breakpoints associated with the amplified DNA. Although next-generation sequencing technology typically involves the generation of short polynucleotide reads (<300 bp) that are ill-suited for the analysis of CNV structure due to the mapping ambiguity of short reads in the presence of highly homologous or repetitive sequences [9], the recent advent of long-read sequencing technologies such as Nanopore allows reads to be uniquely mapped to the reference genome, facilitating a more effective CNV detection and identification of previously cryptic CNV breakpoints [10].

To explore the mechanisms responsible for the formation of CNVs in chromosome 20, we have now used Nanopore long-read next generation sequencing to analyze the local genomic architecture and breakpoints of two examples of a chromosome 20q11.21 CNV, present as a tandem duplication in one PSC line, and as triplication in a second.

## Materials and Methods

### Human PSC culture

The MShef7 [11,12] (hPSCreg) human embryonic stem cell (ESC) line was derived at the University of Sheffield Centre for Stem Cell Biology under the HFEA license R0115-8A (center 0191) and HTA license 22510. A mosaic subpopulation of chromosome 20 variant cells was detected in a culture of MShef7, which was subcloned using single cell deposition by fluorescence-activated cell sorting. The NCRM1 [13] (hPSCreg) human-induced pluripotent stem cell (iPSC) line was acquired from RUCDR Infinite Biologics, and was originally derived by reprogramming umbilical cord blood CD34+ cells using a nonintegrating episomal vector. Both cell lines were maintained in culture vessels coated with a matrix of Vitronectin human recombinant protein (A14700; Thermo Fisher Scientific) and batch fed daily with mTeSR (85850; STEMCELL Technologies). Once the cells had reached confluency, they were passaged using ReLeSR (05873; STEMCELL Technologies) according to manufacturer's guidelines.

### Quantitative polymerase chain reaction breakpoint determination

DNA was extracted from cell pellets using the DNeasy Blood and Tissue kit (69504; Qiagen). DNA quantity and quality were measured using a NanoPhotometer (Implen). One microgram of DNA was digested with 10 U of FastDigest EcoRI enzyme (FD0275; Thermo Fisher Scientific) in FastDigest buffer (FD0275; Thermo Fisher Scientific) for 5 min at 37°C, followed by deactivation of the enzyme by incubating at 80°C for 5 min. Quantitative polymerase chain reaction (qPCR) was performed as previously described [14,15], using the adapted protocol [14], whereby primer sets were designed along the length of the q arm of chromosome 20 (Table 1) to allow an estimate of the amplicon length. A 10-μL PCR contained TaqMan Fast Universal PCR mastermix (4366072; Thermo Fisher Scientific), 0.1 μM Universal probe library hydrolysis probe, 0.1 μM each of the forward and reverse primers (Table 1), and either 20 ng of EcoRI-digested DNA or water only (no template control). The PCRs were run on the QuantStudio 12K Flex Real-Time PCR System using the following profile: 50°C for 2 min, 95°C for 10 min, and 40 cycles of 95°C for 15 s and 60°C for 1 min. The copy number was determined by first subtracting the average Cq values from the test sample 20q loci from the reference loci (Chromosome 4p) to obtain a dCq value. The dCq for the calibrator sample at the same loci was then calculated in the same way, and the test sample dCq and calibrator sample dCq were subtracted from one another to obtain ddCq. The relative quantity was calculated as 2^−ddCq^. Finally, to obtain the copy number, the relative quantity was multiplied by 2.

**Table 1.** Quantitative Polymerase Chain Reaction Breakpoint Detection Primer Sets and Probes [15]

Gene (location) accession no.	Primer sequences (forward and reverse)	UPL probe no.
*RELL1* (4p14) NC_000004.12	tgcttgctcagaaggagctt tgggttcaggaacagagaca	12
*DEFB115* (20q11.21) 31,257,664 NM_001037730.1	tcagcctgaacattctggtaaa cacttgtcttttccccaaactc	14
*REM1* (20q11.21) 31,475,272 NM_014012.5	ccccttttctcactccacaa tctgcagggggagaagtaca	46
*TPX2* (20q11.21) 31,739,101 NM_012112.4	cccccaaatcaggcctac ttaaagcaaaatccaggagtcaa	35
*MYLK2* (20q11.21) 31,819,375 NC_000020.11	ggtcaggagaacccagagtg gtctcccagggcacttcag	16
*XKR7* (20q11.21) 31,968,002 NM_033118.3	gtgtcttaccggggtcctatc gcctggaaggtgtgcagta	3
*TM9SF4* (20q11.21) 32,109,506 NM_014742.3	taatggagccaatgccagta caaaaccagtttctgtgccttt	45
*ASXL1* (20q11.21) 32,358,062 NM_015338.5	gagtgtcactgtggatgggtag ctggcatatggaaccctcac	13

UPL, Universal probe library.

### Fluorescence in situ hybridization for the detection of chromosomal variants

Human PSCs were detached from culture flasks by incubating with TrypLE Express Enzyme (11528856; Fisher Scientific) for 3 min at 37°C. The cells were collected in Dulbecco's modified Eagle's medium/F12 basal media (D6421; Sigma Aldrich) and centrifuged at 270 *g* for 8 min. To the cell pellet, 1 mL of prewarmed 37°C 0.0375 M potassium chloride was added. The cells were then centrifuged at 270 *g* for 8 min, before fixing the cells by adding 2 mL fixative (three parts methanol:one part acetic acid, v/v), in a drop-wise manner under constant agitation. Fluorescence in situ hybridization (FISH) detection of chromosomal variants was performed by Sheffield Diagnostics Genetic Service. Analysis was performed on 100 interphase nuclei per sample that had been probed with RP11-597C24 (BCL2L1) probe (BlueGnome, Illumina) and a telomeric 20p SpectrumGreen (05J03-020; TelVysion) or 20q SpectrumOrange probe (05J04-020; Telvysion).

### DNA extraction for sequencing

DNA was extracted from cell pellets using the DNeasy Blood and Tissue kit (69504; Qiagen). DNA quantity and quality were measured using a NanoPhotometer (Implen).

### DNA sequencing

DNA library preparation was performed using the ligation (SQK-LSK108; Oxford Nanopore Technologies) or Rapid sequencing kits (SQK-RAD004; Oxford Nanopore Technologies) according to the manufacturer's Genomic DNA by Ligation or Rapid Sequencing protocols, respectively. The whole-genome libraries were sequenced using the Oxford Nanopore MinION or GridION sequencers with the R9.4.1 flow cell (FLO-MIN106D; Oxford Nanopore Technologies) following the manufacturer's instructions. Each flow cell yielded ∼5 Gb of data.

### Data processing

Data exported as FASTQ files were mapped to the chromosome 20 hg38 reference sequence using minimap2 sequence aligner (version 2-2.15) [16]. File management, merging, sorting, and indexing were performed using Sambamba (version 0.6.6) and Samtools (version 1.9) [17,18]. Breakpoint regions were inspected manually using integrated genomics viewer (IGV) [19], and the breakpoint location was identified based on read depth and soft-clipped sequence analysis. In brief, the aligned and sorted .bam files were opened using IGV genomic viewer with soft-clipped bases enabled. The distal breakpoint region identified by qPCR was inspected, and the breakpoint at the single nucleotide level was located by identifying a region of reduced read depth with soft-clipped reads that spanned the point of reduced read coverage (Supplementary Fig. S2A, B). To identify the proximal breakpoint, we reasoned that the soft-clipped proportion of the sequencing reads at the distal breakpoint will map to the breakpoint at the proximal breakpoint. Contiguous sequences of the soft-clipped reads were generated using Canu or through manual assembly [20]. We queried the soft-clipped portion of the reads using BLAT sequence alignment to identify the sequence matches in the human reference genome with high similarity. This study utilised MasterShef7 human Embryonic Stem Cell line with an approval by the U.K. Stem Cell Steering Comitee. Human Induced Pluripotent Stem Cell line NCRM1 was certified for use in EU funded projects by the hPSCreg.

## Results

By interphase FISH analysis, the human ESC line MShef7-A4, a subline of MShef7 [11,12], and the human iPSC line NCRM1 [13] each exhibited a homogeneous population of cells with the gain of a segment from the chromosome 20q11.21 region (Supplementary Fig. S1). The amplicons from each cell line were of a different length but both contained the *BCL2L1* gene. In MShef7-A4, the amplicon was present as a duplication, whereas in NCRM1 it was present as a triplication (Supplementary Fig. S1).

**FIG. 1.**
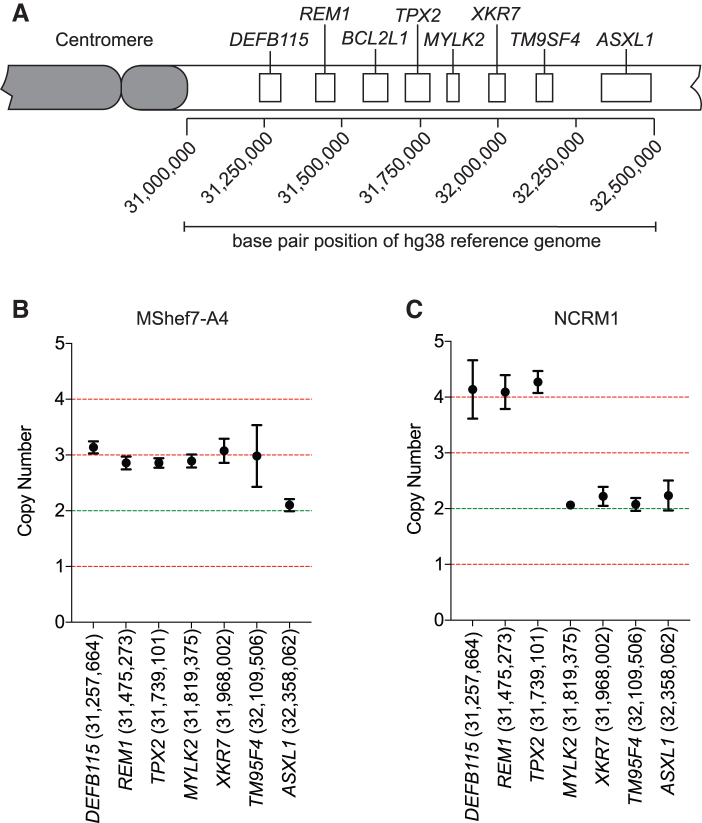
qPCR detection of distal breakpoint positions. **(A)** A schematic showing the position and order of genes probed by qPCR along the chromosome 20q11.21. Primer sets were designed to target intronic regions of the genes displayed. **(B)** Copy number values for the human ESC line MShef7-A4, determined by qPCR for loci along the length of chromosome 20q11.21. The primer locations according to the hg38 reference genome are also displayed with the gene names along the *x*-axis. **(C)** The qPCR determined copy number for loci along the length of chromosome 20q11.21 in the NCRM1 human iPSC line. The copy number of four between *DEFB115* and *TPX2* indicates a triplication of this region. ESC, embryonic stem cell; iPSC, induced pluripotent stem cell; qPCR, quantitative polymerase chain reaction. Color images are available online.

To identify the approximate proximal and distal breakpoint position of the amplicon in each cell line (Fig. 1), we adapted our previously published qPCR-based method for assessment of copy number of target loci, and we used it to assess the copy numbers of loci along the length of the q arm of chromosome 20 [14,15]. In both cell lines, the proximal breakpoint was positioned between the centromere and the *DEFB115* gene (Fig. 1). In MShef7-A4, the distal breakpoint of the tandem duplication was located between the *TM9SF4* and *ASXL1* genes (Fig. 1A, B), whereas in NCRM1 the amplicon was smaller with the distal breakpoint positioned between the *TPX2* and *MYLK2* genes (Fig. 1A, C). In addition to identifying the putative breakpoints at 20q11.21, qPCR analysis revealed the presence of four copies of the amplicon in NCRM1, confirming the triplication of the chromosome 20q11.21 region in this line (Fig. 1C).

**FIG. 2.**
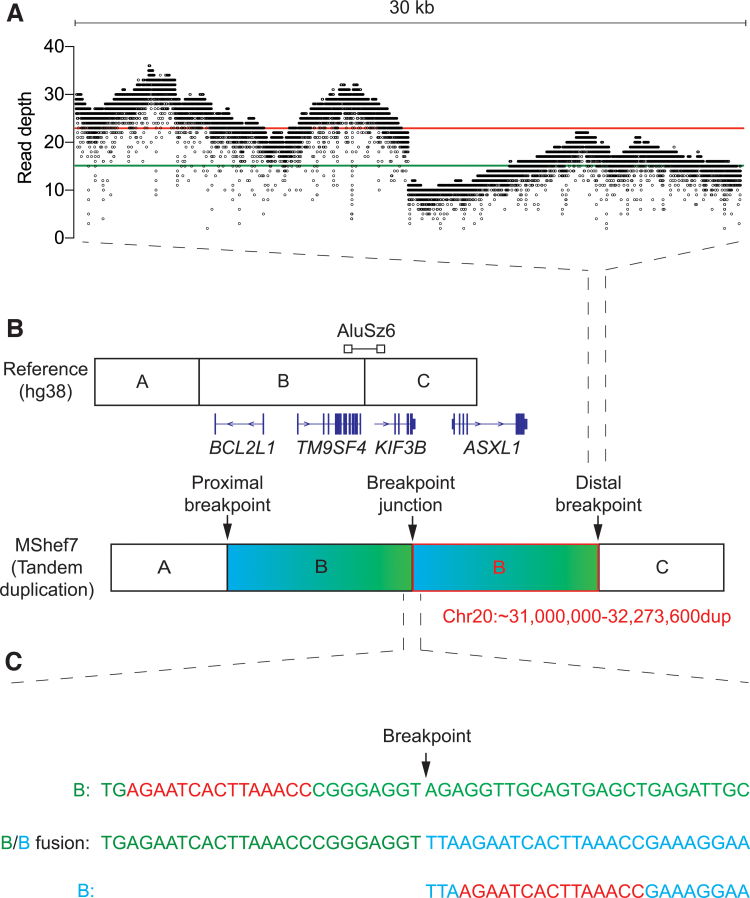
Breakpoint junction detection in MShef7-A4 using Nanopore sequencing. **(A)** Sequencing read coverage of 30 kb spanning the distal breakpoint junction at 32,273,600 bp (chromosome 20q11.21) of the hg38 reference genome. Each *dot* indicates the read depth at a single base pair position. The *red* and *green* lines indicate the mean read depth before and after the breakpoint position, respectively. **(B)** Schematic of the reference genome and the tandem duplication detected in MShef7-A4. Junction between genome segment A-B and B-C represents the proximal and distal breakpoints, respectively. The position of genes flanking and the location of the *AluSz6* in relation to the breakpoint are depicted. **(C)** Reference sequence spanning the distal breakpoint (B—*top*, *green*), sequence of the breakpoint junction (B/B fusion—*middle*), and the contig sequence of the distal side of the proximal breakpoint (B—*bottom*, *blue*). The regions of microhomology that flank the proximal and distal breakpoints are indicated in *red*. Color images are available online.

To identify the location of the breakpoints at a single nucleotide resolution in MShef7-A4 CNV and to determine the orientation of this tandem duplication, we performed whole-genome Oxford Nanopore sequencing on DNA extracted from the cells and aligned the sequencing reads to the hg38 human reference genome assembly [21]. The average read depth across chromosome 20 was 14.5 with a mean read length of 15.2 kb. We noted a 1.57-fold increase in sequencing read depth along the chromosome 20q11.21 relative to the rest of the chromosome (22.8 vs. 14.5, respectively), indicating a change in the copy number of the 20q11.21 region from 2 to 3 (Fig. 2A) [22,23]. A distinct drop in read coverage was observed at position 32,273,600 bp of the chromosome 20 hg38 reference sequence (between *TM95F4* and *ASXL1* genes), which we surmised to be the distal breakpoint, consistent with the approximate position we inferred by qPCR (Fig. 1A and 2A).

To represent reads that map to two discontinuous locations in the genome, mapping algorithms use “soft-clipping” to indicate that a portion of the read in question does not map to the same position as the remainder of the read [17]. Reads that span breakpoints trigger soft clipping because they map to different regions of the reference genome and so provide evidence of structural variation; in our case, tandem duplication (Supplementary Fig. S2) [24,25].

We performed a BLAT pairwise sequence alignment [26] of the unmapped DNA sequence at the breakpoint and identified a (GGAAT)n microsatellite repeat with 92% identity to a pericentromeric region proximal of the *DEFB115* gene, confirming the head-to-tail orientation of the tandem duplication (Fig. 2B, C). This microsatellite is positioned at 31,051,509–31,107,036 bp on chromosome 20, and is flanked by two unmapped regions of the reference genome. We could not locate the proximal breakpoint to a single nucleotide position, which we inferred was due to the breakpoint being located in a currently unmapped region of the reference genome, potentially in one of the regions we observed flanking the microsatellite.

To understand the mechanism of tandem duplication in MShef7-A4, we analyzed the breakpoint sequences for signatures commonly observed in CNVs. From this analysis, we identified a region of microhomology (AGAATCACTTAAACC) that flanked both the proximal and distal breakpoint positions (Fig. 2B, C). By consulting the Dfam database of transposable elements, we observed that the distal region of microhomology lies within an *AluSz6* retrotransposon that spans the distal breakpoint [27]. These results suggest a role of microhomology in the mutational mechanism of the tandem amplification of chromosome 20 in the MShef7-A4 cell line.

**FIG. 3.**
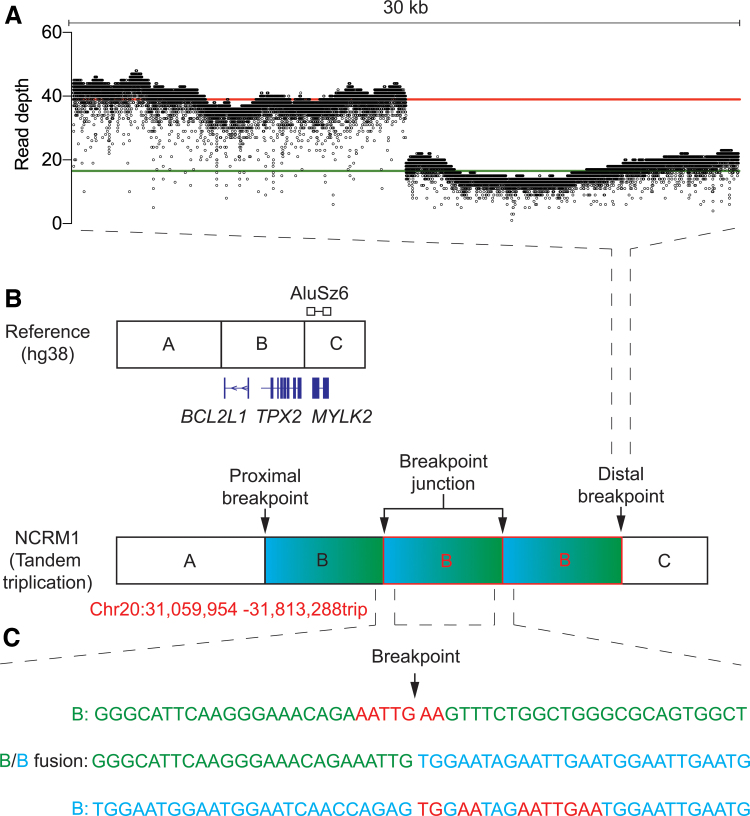
Breakpoint position of the tandem triplication in NCRM1. **(A)** Read coverage of 30 kb surrounding the breakpoint junction 31,813,288 bp (chromosome 20q11.21) of the hg38 reference genome. The mean read depth before and after the breakpoint is shown (*red line* and *green line*, respectively). **(B)** Schematic depicting the reference genome and the NCRM1 tandem triplication. The distal breakpoint lies between the junction of B-C, and the proximal breakpoint is located on the boundary of the A-B segments. The genes flanking the breakpoint, as determined by qPCR, are depicted. The position of the *AluSz6* identified from the Dfam database is represented above the reference sequence schematic. The exact nucleotide position of the proximal and distal breakpoints is written in *red* below the schematic of the tandem triplication. **(C)** Reference sequence spanning the distal breakpoint (B—*top*, *green*), the proximal breakpoint (B—*bottom*, *blue*), and the combined amplification breakpoint junction (B/B fusion—*middle*). The region of microhomology that flanks each of the breakpoints is highlighted (*red*). Color images are available online.

We used the same sequencing approach to identify and analyze the breakpoints in the human iPSC line, NCRM1, which contains a tandem triplication in the 20q11.21 region (Supplementary Fig. S1 and Fig. 1C). Our Nanopore sequencing returned an average read length of 19.9 kb at a mean depth of 20.3 across chromosome 20. The increased read depth associated with CNVs was greater in NCRM1 (2.2-fold) (Fig. 3A) when compared with MShef7-A4, consistent with the presence of 20q11.21 triplication in NCRM1 indicated by our PCR and FISH analyses. In line with our qPCR analysis, long-read sequencing identified a sole distal breakpoint at position 31,813,288 bp between the *TPX2* and *MYLK2* genes.

To identify the proximal breakpoint position, we performed a BLAT pairwise sequence alignment on the unmapped portions of the soft-clipped reads. Our soft-clipped sequence aligned with the reference genome at position 31,059,954 bp, within the same microsatellite that was putatively identified as the proximal breakpoint region in MShef7-A4 (Fig. 3B, C). These data confirm that the tandem triplication of chromosome 20q11.21 in NCRM1 has occurred in a head-to-tail orientation, and that each amplicon was of equal length and contained the same breakpoint positions. Furthermore, we observed a common microsatellite sequence at the proximal breakpoint in both cell lines, and thus, its involvement could be complicit in the tandem amplifications that commonly occur associated with chromosome 20q11.21.

**FIG. 4.**
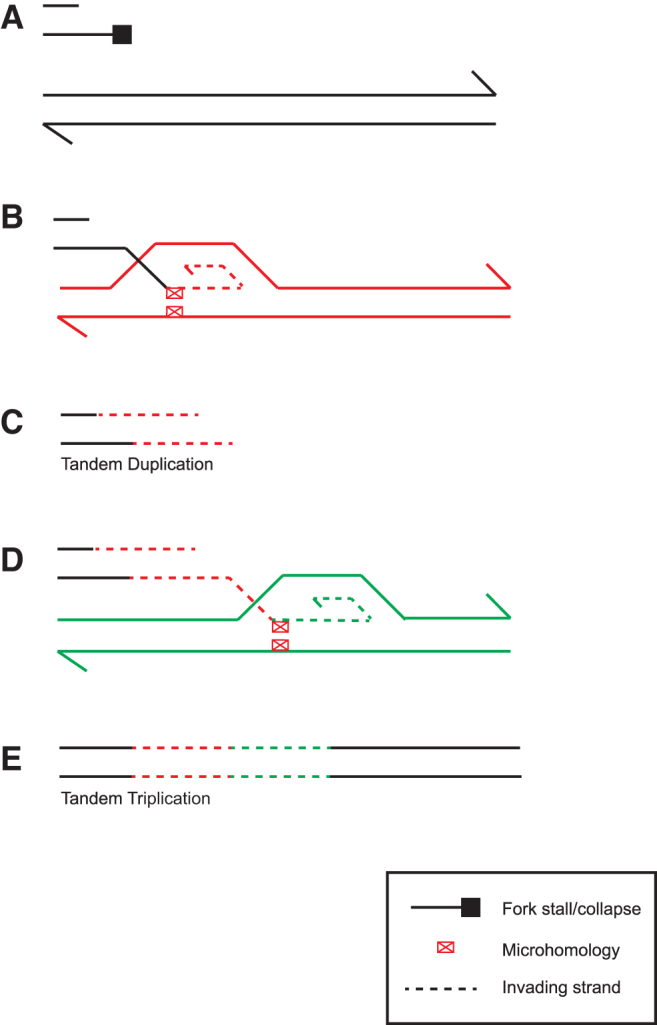
Model for microhomology-mediated tandem amplification in human PSCs. **(A)** Replication fork stalling is promoted by *Alu* sequences that form hairpin loops. **(B)** Repair by microhomology-mediated break-induced replication is initiated by strand invasion at a site of microhomology in the pericentromeric microsatellite on the sister chromatid. **(C)** Replication proceeds, duplicating 20q11.21. **(D)** An additional round of strand invasion and resynthesis occurs in examples of **(E)** tandem triplication. PSC, pluripotent stem cell. Color images are available online.

To infer the mechanism involved in the tandem triplication of chromosome 20q11.21 in NCRM1, we interrogated the reference sequence at both the proximal and distal breakpoint positions. We identified multiple regions of microhomology (TGAA and AATTGAA) that flanked both sides of the fusion junction (Fig. 3C). Furthermore, we consulted the Dfam database [27] of transposable elements and identified an *AluSz6* element that was situated 9 bp downstream of the distal breakpoint (Fig. 3B, C). As we were unable to find an *Alu* element at the proximal breakpoint itself, it is unlikely the tandem duplication and triplication in MShef7-A4 and NCRM1, respectively, have arisen from a mechanism of *Alu-Alu* recombination. Instead, we propose that the *Alu* elements are sites of chromosome fragility, due to replication blockage [28–32]. Repair of stalled and collapsed forks would then proceed through break-induced replication at complementary sites of microhomology (microhomology-mediated break-induced replication), and strand invasion upstream on the same or a homologous chromosome would generate a tandem amplification (Fig. 4).

## Discussion

The Nanopore sequencing that we have described here has allowed us to identify the breakpoints associated with tandem amplifications of chromosome 20q11.21 in two human PSCs, MShef7 and NCRM1. In both cases, the amplicon was arranged in a head-to-tail orientation, and the distal breakpoints are located in or close to *Alu* sequences. The proximal breakpoints of each were located in a pericentromeric microsatellite region close to 31 Mb on chromosome 20. In the case of the iPSC line, NCRM1, which contains the tandem triplication, each amplicon was of equal length with the same breakpoint positions. A detailed characterization of the breakpoints at a single nucleotide level revealed short microhomologies that flank or overlap both the proximal and distal breakpoints.

CNVs typically arise from errors in the repair of genomic damage, such as double-stranded breaks, by mechanisms that include both homologous and nonhomologous recombination events [7]. Evidence of the repair mechanism that has operated on a DNA lesion to generate a CNV can be characterized by analysis of the breakpoint sequences [33,34].

The breakpoints of CNVs formed by nonhomologous end-joining (NHEJ) do not usually exhibit microhomology although, in rare examples, microhomology of between 1 amd 4 bp has been reported [35,36]. As the microhomology at the breakpoints of amplicons in both MShef7-A4 and NCRM1 was >7 bp it is unlikely that classical NHEJ is the mechanism of tandem amplification in the two present cases. Alternative forms of end-joining such as microhomology-mediated end-joining do utilize larger spans of homology or microhomology [37–42]. These mechanisms differ from classical NHEJ, as they do not involve blunt-end ligation but instead utilize end-resection at DNA breaks to reveal overlapping microhomologous single-stranded DNA [43]. Resection of the DNA in this manner creates an insertion of >10 bp [44–46], which were not present in the breakpoints described here.

The tandem amplifications in MShef7 and NCRM1 had breakpoints devoid of large regions of sequence homology, which ruled out mechanisms involving homologous recombination such as nonallelic homologous recombination [47]. However, the presence of an *AluSz6* element at the distal breakpoints in both cell lines led us to consider *Alu-Alu*-mediated nonallelic homologous recombination mechanism. For *Alu-Alu*-mediated nonallelic homologous recombination to take place it would require a second *Alu* element at the proximal breakpoint with high sequence identity with the distal *Alu* [48]. We found no evidence of a second *Alu* at the proximal breakpoint in either of our cell lines.

Despite this, the presence of *AluSz6* at distal breakpoints in both cell lines suggests that it might play a role in the initiation of tandem amplifications, rather than in the mechanism of mutation itself. Inverted repeats, such as *Alu* elements, form hairpin loop secondary structures that can impede replication, leading to fork stalling and collapse, particularly under conditions of replication stress [28–32,49–51]. We have previously reported that during in vitro culture, human PSCs are particularly susceptible to high levels of DNA replication stress, which is also associated with replication fork stalling and collapse [52–54].

The breakpoint signatures of the tandem amplifications characterized in MShef7–A4 and NCRM1 are consistent with the DNA replication-based microhomology-mediated break-induced replication, which are initiated by replication fork stalling and collapse [33,55]. Microhomology-mediated break-induced replication is initiated from the 5′ end of a DNA break at a collapsed fork, and is resected to generate a 3′ single-stranded overhang, which then invades a template region with microhomology before replication is reinitiated. If the template is upstream on the same chromosome or a homologous chromosome, a tandem amplification would result (Fig. 4A–C) [33,47,55,56]. Furthermore, the role of microhomology-mediated break-induced replication in the formation of tandem triplications has been discussed [7,34,55,57]. Should replication fork collapse lead to sister chromatid strand invasion at an upstream region of microhomology, replication of the amplified segment will proceed. This could then be followed by a second round of template switching and strand invasion at the same region of microhomology, although this time into the other parental homolog with replication proceeding to the distal end of the chromosome, resulting in a tandem triplication (Fig. 4).

## Conclusion

Here, we have performed long-read Nanopore sequencing to gain insight into the mechanism that drives recurrent tandem amplification of chromosome 20q11.21 in human PSCs. We identify a common repetitive motif and regions of microhomology that encapsulate the unique breakpoints in two cell lines. Strikingly, a parallel study has identified the same (GGAAT)n at the variable distal breakpoint of 11 further cell lines with 20q11.21 CNVs [58]. Collectively, these findings suggest that this chromosomal region is predisposed to tandem amplification, which is driven by microhomology-mediated break-induced replication [58]. This mechanism is also consistent with the constitutive replication stress to which human PSCs are particularly susceptible during in vitro culture [54]. Associated replication fork stalling and collapse could be exacerbated by *Alu* elements, which might then initiate such mutations at Alu-rich regions of the genome.

The recurrent nature of genetic change in human PSCs is considered nonrandom due to the selection of advantageous mutations. However, it was recently reported that mutations in human PSCs occur with higher frequency in nongenic regions [59]. The data presented here complement these findings, and suggest that mutation itself may be nonrandom but may be enriched at certain sites that can be characterized by the genomic architecture. By defining these regions, it may be possible to safeguard the genome stability of human PSCs for their use in cell-based regenerative medicine.

## Supplementary Material

Supplemental data

Supplemental data
