# Supplementary material for: Nanopore Sequencing Indicates That Tandem Amplification of Chromosome 20q11.21 in Human Pluripotent Stem Cells Is Driven by Break-Induced Replication
Source: Stem Cells Dev. 2021 May 25;30(11):578–86. doi: 10.1089/scd.2021.0013 (PMC8165465; doi:10.1089/scd.2021.0013)
Supplement: Supplemental data [file Supp_FigureS2.doc]

**Supplementary Figure 2 | Breakpoints identification in IGV genomics viewer. A,** Screenshot of the distal breakpoint position in MShef7-A4 (32,273,600 bp). Read depth shows a distinctive drop in coverage at the centre of the image that coincides with soft clipped reads that flank downstream of this position. **B,** IGV image of the distal breakpoint (31,813,288 bp) in NCRM1. The breakpoint was identified from read depth change and soft-clipped read identification. **C,** The soft-clipped reads from the distal breakpoint mapped to 31,059,954 bp. Inset, reference sequence surrounding the breakpoint position.
