# Supplementary material for: Nanopore Sequencing Indicates That Tandem Amplification of Chromosome 20q11.21 in Human Pluripotent Stem Cells Is Driven by Break-Induced Replication
Source: Stem Cells Dev. 2021 May 25;30(11):578–86. doi: 10.1089/scd.2021.0013 (PMC8165465; doi:10.1089/scd.2021.0013)
Supplement: Supplemental data [file Supp_FigureS1.doc]

**Supplementary Figure 1 | Interphase FISH detection of chromosome 20 tandem amplification.** Representative image of interphase FISH performed on MShef7-A4 (**A**) and on NCRM1 (**B**). The white arrow heads indicate the duplication (green) **(A)** and triplication (red) **(B)** of 20q11.21. The cell lines were hybridised with a fluorescently labelled RP11-597C24 BAC 20q11.21 probe (**A**, green. **B**, red) and chromosome 20q telomere probe (red) (**A**) or a chromosome 20p telomere probe (green) (**B**), DNA was co-stained with DAPI (Blue).
